# Effects of the Brown Seaweed *Laminaria japonica* Supplementation on Serum Concentrations of IgG, Triglycerides, and Cholesterol, and Intestinal Microbiota Composition in Rats

**DOI:** 10.3389/fnut.2018.00023

**Published:** 2018-04-12

**Authors:** Jae-Young Kim, Young Min Kwon, In-Sung Kim, Jeong-A. Kim, Da-Yoon Yu, Bishnu Adhikari, Sang-Suk Lee, In-Soon Choi, Kwang-Keun Cho

**Affiliations:** ^1^Department of Animal Resources Technology, Gyeongnam National University of Science and Technology, Jinju, South Korea; ^2^Department of Poultry Science, University of Arkansas, Fayetteville, AR, United States; ^3^Cell and Molecular Biology Program, University of Arkansas, Fayetteville, AR, United States; ^4^Department of Animal Science and Technology, Sunchon National University, Suncheon, South Korea; ^5^Department of Biological Sciences, Silla University, Busan, South Korea

**Keywords:** brown seaweed, experimental model, intestinal microbiota, *Laminaria japonica*, pyrosequencing

## Abstract

The intestinal microbial communities play critical roles in various aspects of body function of the host. Prebiotics, such as dietary fiber, can affect health of the host by altering the composition of intestinal microbiota. Although brown seaweed *Laminaria japonica* is rich in dietary fiber, studies on its prebiotic potential are quite rare. In this study, basal diet (control), basal diet supplemented with dried *L. japonica* (DLJ), heat-treated dried *L. japonica* (HLJ), or heated dried *L. japonica* with added fructooligosaccharide (FHLJ) was fed to rats for 16 weeks. Serum concentrations of IgG, triglyceride, and cholesterol were measured. In addition, the intestinal microbiota composition was analyzed by high-throughput sequencing of 16S rRNA gene. As compared to the control group, DLJ, HLJ, and FHLJ groups showed significantly higher serum IgG concentration, but had lower weight gain and serum triglyceride concentration. Moreover, DLJ, HLJ, and FHLJ groups showed lower *Fimicutes* to *Bacteroidetes* ratio when compared with the control group. As compared with the control group, obesity-associated bacterial genera (*Allobaculum, Turicibacter, Coprobacillus, Mollicute*, and *Oscilibacter*), and the genera with pathogenic potentials (*Mollicute, Bacteroides, Clostridium, Escherichia*, and *Prevotella*) decreased while leanness-associated genera (*Alistipes, Bacteroides*, and *Prevotella*), and lactic acid bacterial genera (*Subdoligranulum, Streptococcus, Lactobacillus, Enterococcus*, and *Bifidobacterium*) increased in all treatment groups. On the contrary, butyric acid producing genera including *Subdoligranulum, Roseburia, Eubacterium, Butyrivibrio*, and *Anaerotruncus* increased significantly only in FHLJ group. The overall results support multiple prebiotic effects of seaweed *L. japonica* on rats as determined by body weight reduction, enhanced immune response, and desirable changes in intestinal microbiota composition, suggesting the great potential of *L. japonica* as an effective prebiotic for promotion of host metabolism and reduction of obesity in humans.

## Introduction

Unlike cellulose contained in vegetables, carbohydrates in seaweeds have multiple beneficial effects to human bodies, including improving intestinal activities with their dietary fibers, discharging heavy metals in foods, and reducing blood lipid concentrations (1, 2). Brown seaweed *Laminaria japonica* contains water-soluble dietary fibers (32.8%) that are highly bioactive in human bodies, and non-water-soluble dietary fibers (17.9%). The total dietary fiber contents in *L. japonica* is therefore 50.7%, which is the highest among all plants and seaweeds (3). The intake of *L. japonica* that has such high dietary fiber contents can inhibit the growth of pathogenic bacteria and promote the growth of beneficial bacteria, suggesting a great prebiotic potential of *L. japonica* (4).

Prebiotics promote the growth of beneficial bacteria, such as lactic acid bacteria, and inhibit the growth of bacteria with pathogenic potentials in the large intestine. In particular, fructooligosaccharide (FOS) is a well-characterized prebiotics, which was shown to promote selective growth of bifidobacteria in the large intestine (5).

Bacteria in the gut microbiota play crucial roles in the overall body function of the host, including host metabolism. It was previously reported that the gut microbiota of the patients with metabolic syndromes such as obesity, arteriosclerosis, or type 2 diabetes are different from those of healthy individuals (6). In addition to the host metabolism, the gut microbiota are also functionally linked to the immune responses and immune systems of the host (7, 8). Multiple factors, including diet, age, and antibiotics, have been demonstrated to influence the maintenance and shaping of the gut microbiota, thereby health status of the host (9–11).

Our previous study conducted using rats reported an increase in beneficial intestinal microbes following the intake of seaweeds *Undaria pinnatifida* and *L. japonica* (4). We were interested in further exploring the prebiotic potential of *L. japonica*. Therefore, this study was conducted to investigate the effects of *L. japonica* on rats when supplemented in diet as dried (DLJ), heat-treated (HLJ), or heat-treated form with added FOS (FHLJ) on body weight, serum concentration of IgG, triglycerides, and cholesterol, and intestinal microbiota composition.

## Materials and Methods

### Animal Experiment

Dried *L. japonica* powder was purchased from Haeormbio Co., Ltd. (Busan, Korea). The heat-treated *L. japonica* was prepared by heating dried *L. japonica* powder for 30 min at 100°C. The FOS used in the experiment was also purchased (OGMayTech Co., Ltd., Seoul, Korea).

Forty eight 6-week-old male Sprague-Dawley rats were purchased from Samtako (Osan, Korea). The rats were subjected to a basal diet (Control), the basal diet mixed with 10% dried *L. japonica* powder (DLJ), the basal diet mixed with 10% heat-treated *L. japonica* (HLJ), or the basal diet mixed with 10% heat-treated *L. japonica* and 0.6% FOS (FHLJ). The compositions of the diets for the control and treatment groups are shown in Table S1 in Supplementary Material. A total of 12 rats were used in each treatment group, which were further divided into 4 repetitions of 3 rats per cage. After being purchased, the rats underwent a 1-week acclimatization period before being subjected to the aforementioned diets for 16 weeks. The weight and feed intake of each rat were measured once weekly throughout the experimental period using all animals (12 rats/group). The animal housing was maintained at the temperature of 22 ± 3°C with relative humidity of 65 ± 5%, and light and dark cycles of 12 h. The feed and drinking water were provided *ad libitum*. At the end of the experiment, the animals were sacrificed, and the ceca and blood were collected from each rat. Cecal samples (four rats per group) were processed and analyzed individually as shown below for microbiome analysis. Serum samples were separated from the blood (four rats per group) to measure the concentrations of serum IgG, cholesterol, and triglyceride as previously described (4). All animal handling procedures were approved by the Institutional Animal Care and Use Committees at Gyeongnam National University of Science and Technology.

### DNA Extraction and PCR

The genomic DNA of intestinal contents was extracted using ZR Fecal DNA Mini Prep™ kit (Zymo Research, USA). The V1–V3 region of 16S rRNA gene was amplified using V1-9F (5′-CCTATCCCCTGTGTGCCTTGGCAGTCTCAGACGAGTTTGATCMTGGCTCAG-3′) and V3-541R (5′-CCATCTCATCCCTGCGTGTCTCCGACTCAG-barcode-ACWTTACCGCGGCTGCTGG-3′) as previously described (4). The amplified products were purified using QIAquick PCR purification kit (Qiagen, Valencia, CA, USA) which was followed by quantification using PicoGreen dsDNA quantitation assay kit (Invitrogen, Carlsbad, CA, USA). The amplicons of individual samples were combined together at equimolar concentration, which was then sequenced using a Roche/454 FLX system (ChunLab, Seoul, Korea).

### Data Analysis

The pyrosequencing sequences of 16S rRNA gene were processed using Java-based multi-step bioinformatics pipeline. Unidirectional sequencing reads were identified with the help of unique barcodes in each individual read. Low-quality sequences (≤300 bp) were removed, and the trimmed reads were clustered at 97% sequence similarity level to pick operational taxonomic units [OTUs; (12)]. For taxonomic identification, representative sequences were selected from the OTUs, and their taxonomy were assigned based on the top five BLASTN hits in the EzTaxon-e database (13). The sequences that did not match in BLASTN searches in the EzTaxon-e database were classified as non-target sequences and were excluded from further analysis. The similarity between the query and candidate species was calculated using the Myers and Miller method (14). The cladogram was calculated using the TBC clustering algorithm (12). The read numbers in each sample were normalized by random subsampling. Overall analysis for phylogenetic differences was performed using the CL community program provided by ChunLab (Seoul, Korea). Statistical significance in relative abundance among different groups was calculated through variance analysis using General Linear Model procedure of the SAS (Ver. 9.1) and means comparison using Duncan’s multiple range tests at 5% significance level.

## Results

### Body Weight Gain and Feed Intake

**Figure 1:**
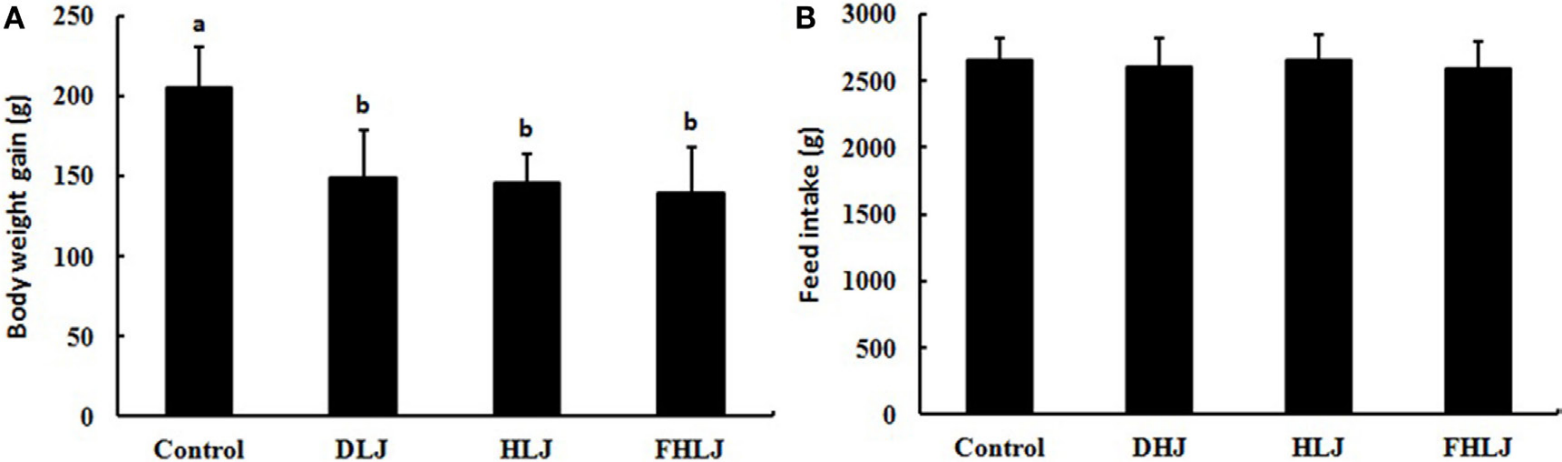
Effects of the consumption of the diets supplemented with *Laminaria japonica* and fructooligosaccharide (FOS) mixture for 16 weeks on **(A)** body weight gain and **(B)** feed intake of rats (*n* = 12 per group). Control: basal diet group, DLJ: basal diet + 10% dried *L. japonica*, HLJ: basal diet + 10% dried *L. japonica* heat-treated at 100°C for 30 min, FHLJ: HLJ + 0.6% FOS. Values are mean ± SD. No common superscripts on the bars indicate significant difference (*p* < 0.001).

The total weight gain was significantly lower in all three treatment groups (DLJ, HLJ, and FHLJ) when compared with the control group; however, no significant differences was observed among treatment groups as shown in Figure 1A. There was no significant difference in the total feed intake among all groups (Figure 1B), suggesting that the weight loss in treatment groups was not due to the reduced feed intake, but due to the consumption of *L. japonica-*supplemented diets. However, we cannot exclude the possibility that the less amount of energy and nutrient contents in the diets for the treatment groups (DLJ, HLJ, and FHLJ) when compared with the control group (Table S1 in Supplementary Material) could have contributed partially to the weight loss.

### Serum IgG and Triglyceride Concentration

**Figure 2:**
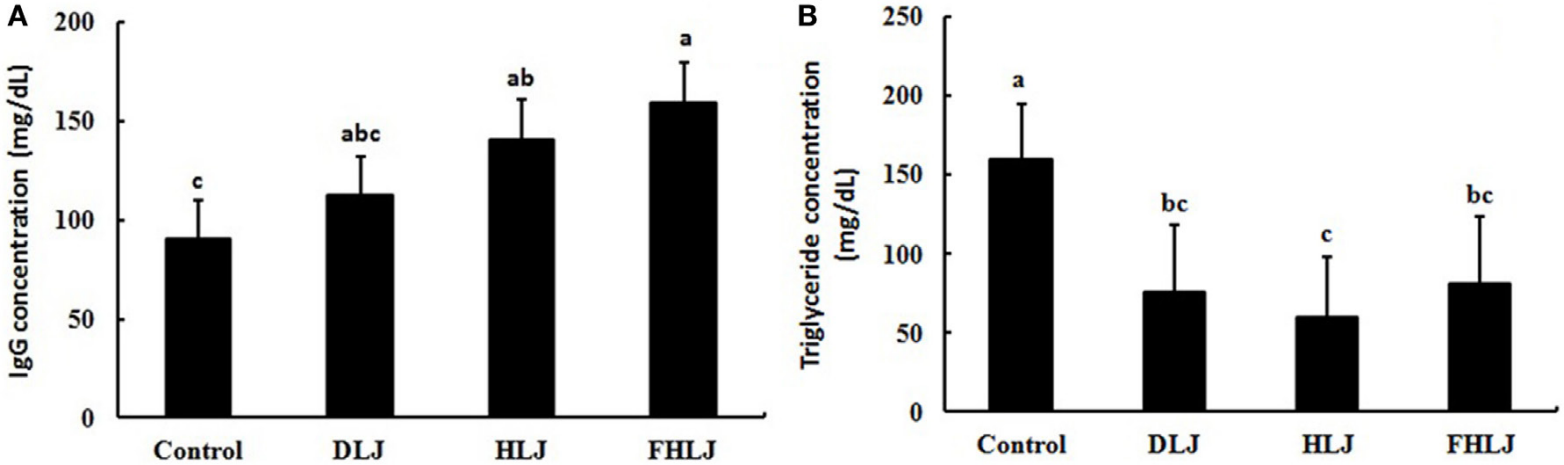
Effects of the consumption of the diets supplemented with *Laminaria japonica* and fructooligosaccharide (FOS) mixture for 16 weeks on **(A)** IgG and **(B)** triglyceride concentrations in the serum of rats (*n* = 4 per group). Control: basal diet group, DLJ: basal diet + 10% dried *L. japonica*, HLJ: basal diet + 10% dried *L. japonica* heat-treated at 100˚C for 30 min, FHLJ: HLJ + 0.6% FOS. Values are mean ± SD. No common superscripts on the bars indicate significant difference in IgG concentration at *p* < 0.05 and in triglyceride concentration at *p* < 0.01.

As compared to the control group, serum IgG concentration was greater in both HLJ and FHLJ groups (Figure 2A). The serum concentration of high-density lipoprotein cholesterol and low-density lipoprotein cholesterol were not different across all treatment groups (data not shown). However, as compared to the control group, serum triglyceride concentration was lower significantly in all treatment groups (Figure 2B). Among the treatment groups, the HLJ group showed the lowest serum triglyceride concentration, although the differences were not significant. Thus, the supply of *L. japonica*, heat-treated *L. japonica*, and heat-treated *L. japonica* with added FOS increased serum IgG concentrations and reduced serum triglyceride concentrations in the experimental animals used in this study.

### Analysis of Intestinal Microbiota

**Figure 3:**
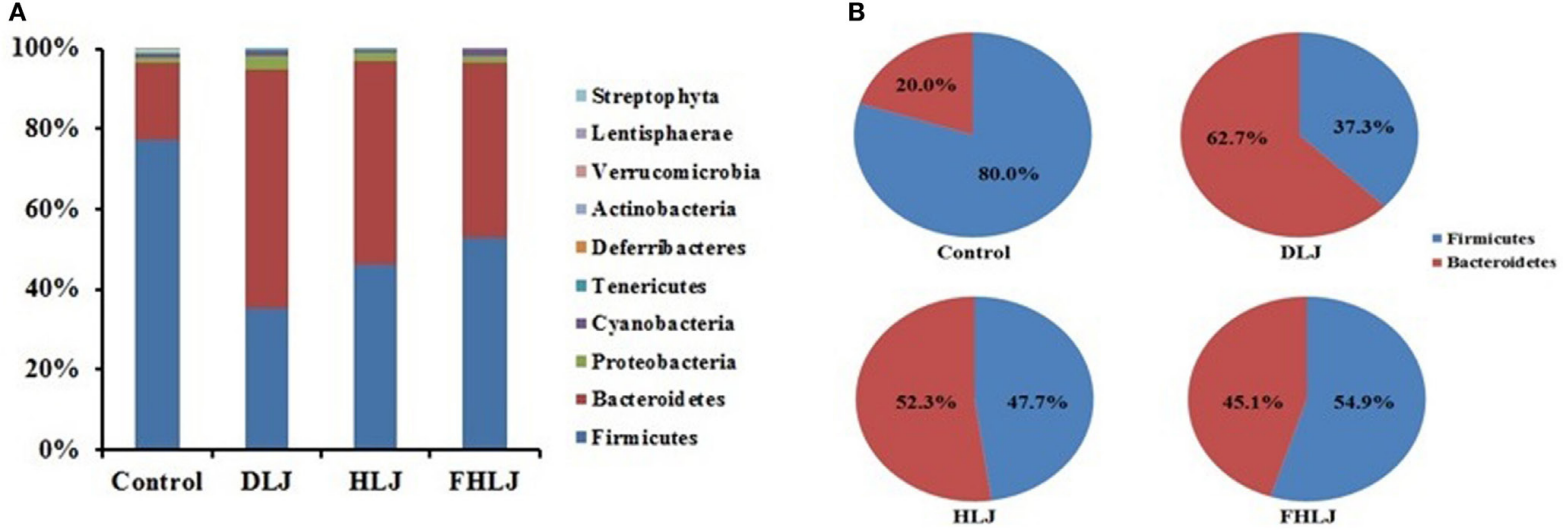
The relative abundance of different phyla in the cecal microbiota of rats (*n* = 4 per group). **(A)** The bar graph shows the relative abundance of 10 phyla, and **(B)** the pie graph shows only two dominant phyla, *Firmicutes* and *Bacteriodetes*, highlighting the changes in the ratio of the two phyla in DLJ, HLJ, and FHLJ in comparison to the control. Control: basal diet group, DLJ: basal diet + 10% dried *L. japonica*, HLJ: basal diet + 10% dried *L. japonica* heat-treated at 100°C for 30 min, FHLJ: HLJ + 0.6% fructooligosaccharide.

Using high-throughput pyrosequencing of 16S rRNA gene, the composition of intestinal microbiota was analyzed using four replicates per group. At phylum level, *Firmicutes* and *Bacteroidetes* were found as dominant phyla since they altogether accounted for at least 95% of total sequence reads in all groups (Figure 3A). The decrease in *Firmicutes* and increase in *Bacteroidetes* were observed in all treatment groups (DLJ, HLJ, and FHLJ) when compared with the control group (Figure 3A). Comparing the proportions of *Firmicutes* and *Bacteroidetes* by normalizing the combined reads of both phyla to 100%, *Firmicutes* was 80.0% in the control, 37.3% in DLJ, 47.7% in HLJ, and 54.9% in FHLJ group (*p* < 0.01). On the contrary, *Bacteroidetes* was 20.0% in the control, 62.7% in DLJ, 52.3% in HLJ, and 45.1% in FHLJ group (*p* < 0.01) (Figure 3B). Among the treatment groups, difference between *Firmicutes* and *Bacteroidetes* was greatest in DLJ group.

**Figure 4:**
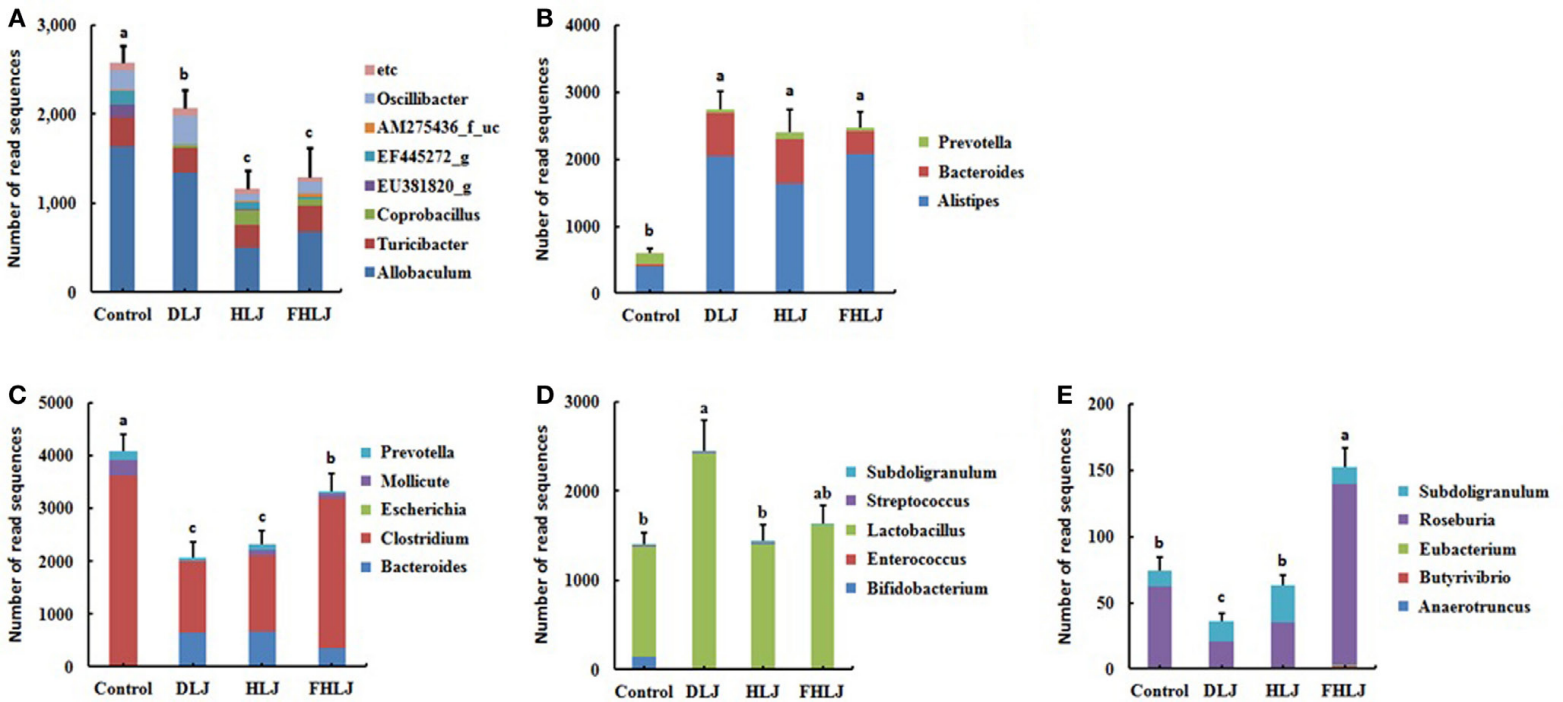
The relative abundance of the functional bacterial groups in the cecal microbiota of rats (*n* = 4 per group). **(A)** Obesity-associated genera, **(B)** leanness-associated genera, **(C)** genera with pathogenic potentials, **(D)** genera that belong to lactic acid bacteria, and **(E)** butyric acid producing genera. Control: basal diet group, DLJ: basal diet + 10% dried *Laminaria japonica*, HLJ: basal diet + 10% dried *L. japonica* heat-treated at 100˚C for 30 min, FHLJ: HLJ + 0.6% fructooligosaccharide. Values are mean ± SD of the numbers of sequence reads of all OTUs belonging to each functional group. No common superscripts on the bars indicate significant difference in the relative abundance of **(A)** obesity-related genera, **(D)** genera that belong to lactic acid bacteria, and **(E)** butyric acid producing genera at *p* < 0.05, and **(B)** leanness-associated genera and **(C)** genera with pathogenic potentials at *p* < 0.01.

According to the functional characteristics of different genera, the microbiome data were further analyzed to determine the relative abundance of the following five functional bacterial groups at genus level: these functional groups included (i) the obesity-associated genera (11, 15), (ii) the leanness-associated genera (16, 17), (iii) the genera with pathogenic potentials (18–20), (iv) the genera belonging to lactic acid bacterial (LAB) (21, 22), and (v) the butyric acid producing genera (21, 23) (Figure 4). The relative abundance levels of these functional groups were indicated with the combined sequence read numbers of the OTUs corresponding to the genera that belong to each functional group (Figure 4). We reasoned that changes in the abundance of these functional groups might give better insights for correlating the changes in microbiota and other measurements, particularly body weight and serum triglyceride concentration when compared with the abundance changes in conventional taxonomic groups.

The obesity-associated genera including *Allobaculum, Turicibacter*, and *Oscillibater* were found in all groups, and they were significantly decreased in all treatment groups (DLJ, HLJ, and FHLJ) when compared with the control group (Figure 4A). Interestingly, even among the treatment groups, the level of the obesity-associated genera was significantly lower in HLJ and FHLJ as compared to DLJ (Figure 4A). Among the leanness-associated genera including *Alistipes, Bacteroides*, and *Prevotella, Alistipes* was dominant in all groups (Figure 4B). As compared to the control group, all treatment groups showed significant increase in the abundance of these genera. Particularly, *Alistipes* increased at least four-folds and *Bacteroides* increased at least 10 times in all treatment groups when compared with the control group (Figure 4B). The genera with pathogenic potentials, which includes *Bacteroides, Clostridium, Escherichia, Mollicute*, and *Prevotella*, were found in all groups except HLJ in which *Escherichia* was absent (Figure 4C). Within this functional group, *Clostridium* was the dominant genus that accounted for 60–87% sequence reads in all groups. The relative abundance of the genera with pathogenic potentials was significantly lower in all treatment groups (DLJ, HLJ, and FHLJ) when compared with the control group. The abundance of this functional group was even lower significantly in DLJ and HLJ groups when compared with FHLJ group (*p* < 0.01).

When it comes to the genera that belong to LAB, *Bifidobacterium, Enterococcus, Lactobacillus, Streptococcus*, and *Subdoligranulum* were found in all groups, except that *Enterococcus* was not found in DLJ and FHLJ groups (Figure 4D). Among LAB, *Lactobacillus* was the dominant genus in all groups, which was 87% in the control group, and at least 97% in all treatment groups (DLJ, HLJ, and FHLJ). This functional group increased significantly in DLJ group (*p* < 0.05), while the increase was not significant in HLJ and FHLJ when compared with the control group.

For the butyric acid producing genera, there were dramatic differences across different treatment groups. *Anaerotruncus, Roseburia*, and *Subdoligranulum* were found in the control group. In the treatment groups, *Roseburia* and *Subdoligranulum* were found in all treatment groups, while *Butyrivibrio* and *Eubacterium* were additionally found in FHLJ group (Figure 4E). As compared to the control group, total butyric acid producing genera were significantly higher in FHLJ group (*p* < 0.01).

To gain more insights on the role of microbiota in mediating the effects of the seaweed supplementation on body weights (Figure 1A), serum IgG concentrations (Figure 2A), and serum triglyceride concentrations (Figure 2B), we further examined the microbiome data to understand the dynamic changes in the microbiota at species level. We found the three species, such as FJ880918_s, *Alistipes_us*, and *Bacteroides eggerthii*, exhibited most dramatic changes in response to the treatments.

**Figure 5:**
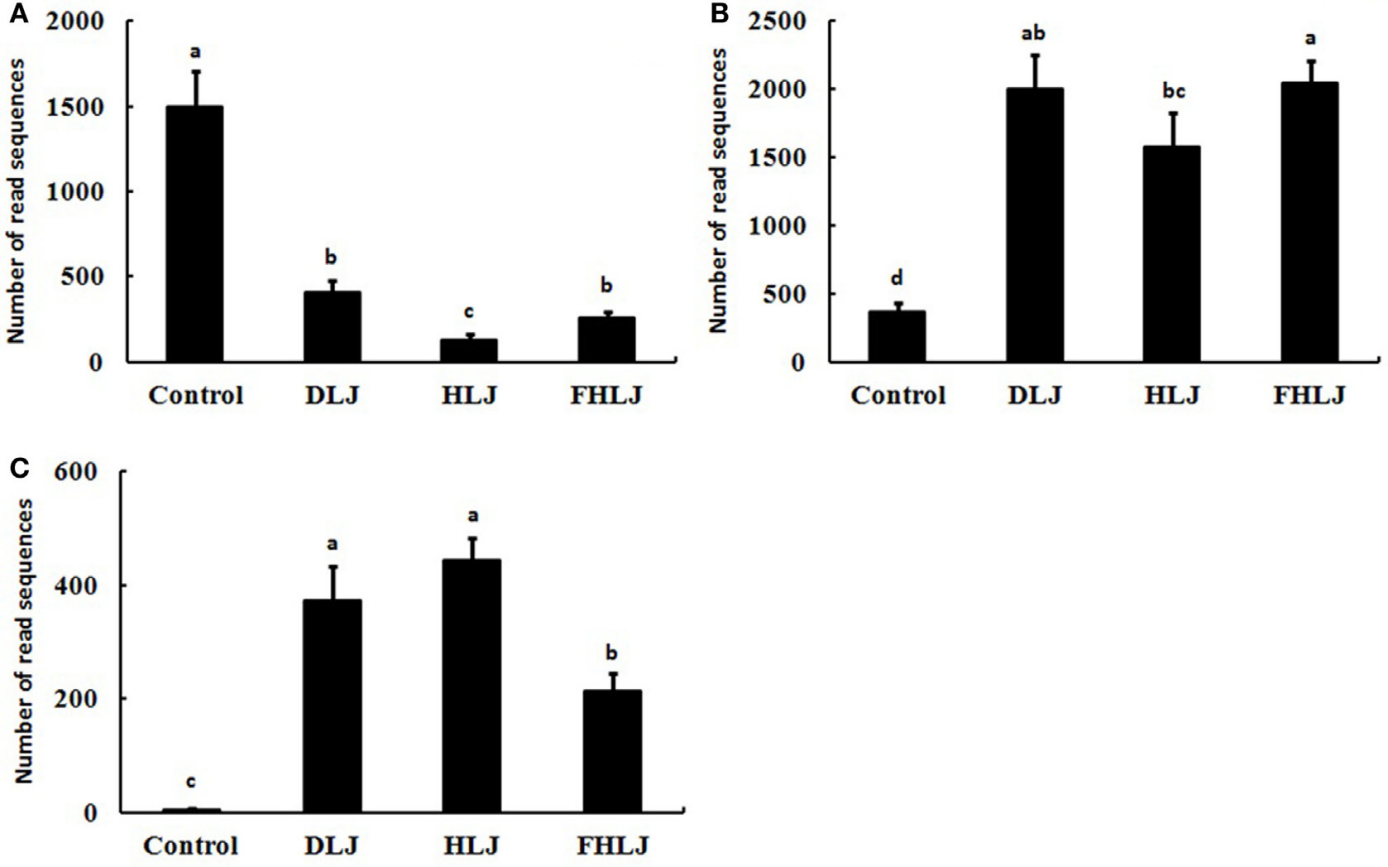
The relative abundance of the bacterial species of interest in the cecal microbiota of rats (*n* = 4 per group). Sequence read numbers of **(A)** *FJ880918_s*, **(B)** *Alistipes_us*, and **(C)** *Bacteroides eggerthii*. Control: basal diet group, DLJ: basal diet + 10% dried *Laminaria japonica*, HLJ: basal diet + 10% dried *L. japonica* heat-treated at 100°C for 30 min, FHLJ: HLJ + 0.6% fructooligosaccharide. Values are mean ± SD of the numbers of sequence reads of all OTUs belonging to each species. No common superscripts on the bars indicate significant difference in **(B)** *Alistipes us* at *p* < 0.05, and **(A)** *FJ880918_s* and **(C)** *Bacteroides eggerthii* at *p* < 0.01.

Species FJ880918_s that belong to genus *Allobaculum*, which was one of the obesity-associated genera shown in Figure 4A, decreased significantly (*p* < 0.01) in the DLJ, HLJ, and FHLJ groups (by 27.5, 8.9, and 17.2%, respectively) when compared with the control group. This species FJ880918_s showed the highest decrease as a single species by the treatments (Figure 5A). The species *Alistipes_us* belongs to genus *Alistipes*, which was a dominant member of the leanness-associated genera as shown in Figure 4B. The relative abundance of *Alistipes_us* was at least 93% within the genus *Alistipes* for all groups, and was 5.37-, 4.23-, and 5.48-fold greater in DLJ, HLJ, and FHLJ groups, respectively, showing significant increase, when compared with the control group (*p* < 0.05; Figure 5B). The species *Bacteroides eggerthii* increased in DLJ, HLJ, and FHLJ groups significantly (by 74.6-, 88.6-, and 42.8-folds, respectively) when compared with the control group (*p* < 0.01; Figure 5C).

## Discussion

Seaweeds are a source of abundant dietary fibers, and *L. japonica* contains the highest dietary fibers among all seaweeds and vegetables (3). Dietary fibers cannot be digested by the digestive enzymes in the stomach and the small intestine, and thus bypass to the large intestine where they are fermented by anaerobic microorganisms (24). Therefore, *L. japonica* has a great potential to be used for effective prevention and treatment of metabolic syndrome caused by high-calorie intakes. Although the efficacy of *L. japonica* has been reported in previous researches (4), limited information is available on the effect of this seaweed on the composition and structure of intestinal microbiota. Our previous study reported reduced weight gain, and proliferations of the leanness-associated bacterial genera as well as other beneficial intestinal bacteria in rats fed with a diet supplemented with *U. pinnatifida* and *L. japonica* (4). This study was aimed at investigating the dietary effects of *L. japonica* in different forms and variations (DLJ, HLJ, and FHLJ) on intestinal microbiota using rats as an animal model. It has been well established that fiber intake is inversely associated with body weight (25). Dietary fibers produce short-chain fatty acids (SCFAs) through fermentation by anaerobic microorganisms in the large intestine (26). Among the SCFAs, butyric acid is used as energy source by intestinal epithelial cells and the remaining SCFAs are absorbed into bloodstream to promote lipolysis by fat cells, control gut hormones, and inhibit fat accumulation by insulin (27–29). In this study, we demonstrated that the supplementation of *L. japonica* (DLJ and HLJ) and FOS-added *Laminaria japonica* (FHLJ) significantly reduced weight gain and serum triglyceride concentrations in rats without affecting feed intakes (Figures 1A and 2B).

Prebiotics have several beneficial effects, such as preventing allergic diseases, regulating immunity, and decreasing the risks of cancers (30–32). The intake of red seaweed *Chondrus crispus* increased blood IgA and IgG concentrations in weaning rats (33). We also reported increase in serum IgG concentrations in all treatment groups (DLJ, HDJ, and FHDJ), indicating that *L. japonica* can enhance immune responses in rats (Figure 2A).

In previous studies, when the intestinal microorganisms of an obese mouse was transplanted into a germ-free mouse, the fat accumulation in germ-free mouse remarkably increased, suggesting intestinal microbes are important players in modulation of obesity (11). *Firmicutes* and *Bacteroidetes* are two major phyla that can affect obesity. Increased level of *Firmicutes* was associated with obesity, while *Bacteroidetes* was shown to promotes leanness (34). In our study, *Firmicutes* and *Bacteroidetes* were dominant phyla in all groups, and *Firmicutes* decreased while *Bacteroidetes* increased when rats were fed with *L. japonica-*supplemented diets (Figure 3). The intake of *L. japonica* (DLJ and HLJ) and FOS-added *L. japonica* (FHLJ) reduced the obesity-associated genera. *Allobaculum, Turicibacter*, and *Coprobacillus* are the genera that belong to the class *Erysipelotrichi* which was found to increase in obese body types (15). *EU381820_g, EF445272_g*, and *AM275436_f_uc* are the genera that fall under the class *Mollicutes*. *Mollicutes* and *Oscillibacter* were shown to increase in obese mouse fed with high-fat diets, which then caused mild inflammations to affect the insulin pathway leading to fat accumulation (11, 35). *L. japonica* (DLJ and HLJ) and FOS-added *L. japonica* (FHLJ) reduced the relative abundance of obesity-associated genera in rat intestine (Figure 4A). *FJ880918s* that falls under the genus *Allobaculum* showed the highest decrease as a single species, suggesting the need for further investigation on this species in future researches (Figure 5A).

Higher abundance of *Prevotella* and *Bacteroides* were reported in African children, who mainly eat typical vegetarian diet when compared with European children, who mainly eat high-fat high-calorie diet (16). Both genera contain microbes that can hydrolyze cellulose and xylan. Mice with higher intestinal abundance of *Prevotella* showed reduction in fat accumulation (36). *Alistipese* that belong to the phylum *Bacteroidetes* was also found to increase in rat with reduced weight gains (37). In this study, the increase in *Alistipes_uc* and *Bacteroides eggerthii* are reported in accordance with the increase in the genera *Alistipes* and *Bacteroides* (Figures 5B,C). Further research is necessary to investigate the interactions between these two species and the host. As compared to the control group, leanness-associated bacterial genera increased in the treatment group supplied with *L. japonica* (DLJ, HLJ, and FHLJ; Figure 4B). Intestinal pathogenic microbes affect immune responses and metabolic status in the hosts. Intestinal pathogenic microbes can also cause mild-grade inflammations. Mild-grade inflammations that appear as immune reactions affect metabolic signaling pathways to promote obesity (38, 39). High-fat diets increase intestinal pathogenic bacteria that induce the expression of cytokines, which are inflammatory substances, and the permeability of the intestine (40). Endotoxins secreted by pathogenic bacteria can cause inflammations due to the activity of macrophages (41). These inflammations increase the expression of TNF-α and NF-κB, and affect the secretion of metabolic hormones, such as insulin, adiponectin, leptin, and resistin, promoting obesity (38, 42). In our study, all treatment groups supplemented with *L. japonica* (DLJ, HLJ, and FHLJ) showed significant decrease in the genera with pathogenic potentials (Figure 4C).

Prebiotics promote the proliferation of intestinal LAB. LAB can inhibit the growth of pathogenic microbes by lowering pH and secreting bacteriocins, as well as can affect immune modulation by increasing concentrations of IgA and γ-interferon in blood (43). It was previously reported that *Bifidobacterium* and *Lactobacillus* in the intestinal microbiota and IgA and IgG increased in rats supplied with red seaweeds (33). *Bifidobacterium* and *Lactobacillus* were also shown to reduce blood cholesterol and lipid components (44). The LAB that have diverse physiological functions increased in all treatment groups supplied with *L. japonica* (DLJ, HLJ, and FHLJ), although the increase was significant only in DLJ when compared with the control group (Figure 4D).

Dietary fibers are fermented by anaerobic microorganisms in the large intestine to produce SCFAs. Butyric acid inhibits insulin’s fat accumulation signaling through SCFA receptor GPR43, relieves inflammations, and reduces liver fat, cholesterol, and triglycerides concentrations (29, 45). In addition, butyric acid generates glucagon-like peptide 1 to stimulate satiety (46). In our study, butyric acid producing bacteria increased only in FHLJ group (Figure 4E).

In summary, the supply of dried *L. japonica* (DLJ), heat-treated *L. japonica* (HLJ), and FOS-added heat-treated *L. japonica* (FHLJ) was found to reduce obesity by lowering weight gain and affect immune modulation by increasing serum concentration of IgG. In addition, they were found to reduce the ratio of *Fimicutes* to *Bacteroidetes*, and the genera with pathogenic potentials, while increasing leanness-associated bacterial genera and LAB. Overall, the effects of dried *L. japonica*, heat-treated *L. japonica*, and FOS-added heat-treated *L. japonica* on intestinal microbiota composition were not significantly different from each other. Therefore, the results of this animal study support the great potential of brown seaweed *L. japonica per se* (without heat treatment or FOS addition) as an effective functional food in humans with beneficial prebiotic effects.

It is important to note that the less amount of energy and nutrient contents in the diets for the treatment groups (DLJ, HLJ, and FHLJ) in comparison with the control group could have contributed partially to the modulation of cecal microbiomes. In the future studies, an improved experimental design employing appropriate inert filler in place of 10% *L. japonica* will be desirable to attribute the microbiome changes completely to the prebiotic effects of different forms of *L. japonica*.

## Ethics Statement

All procedures were approved by the Institutional Animal Care and Use Committees at Gyeongnam National University of Science and Technology (No. 2015-10).

## Author Contributions

J-YK drafted the manuscript and designed the experiments; YMK jointly led the study and revised the manuscript; I-SK, J-AK, and D-YY performed the experiments; BA revised the manuscript; S-SL, I-SC, and K-KC collected and analyzed the data. All the authors read and approved the final manuscript.

## Conflict of Interest Statement

The authors declare that the research was conducted in the absence of any commercial or financial relationships that could be construed as a potential conflict of interest.
