# Supplementary material for: Effects of the Brown Seaweed Laminaria japonica Supplementation on Serum Concentrations of IgG, Triglycerides, and Cholesterol, and Intestinal Microbiota Composition in Rats
Source: Front Nutr. 2018 Apr 12;5:23. doi: 10.3389/fnut.2018.00023 (PMC5906548; doi:10.3389/fnut.2018.00023)
Supplement: Supplementary file 1 [file table_1.docx]

| Items | Treatment | | | | | | | | | | | | | |
| --- | --- | --- | --- | --- | --- | --- | --- | --- | --- | --- | --- | --- | --- | --- |
|  | Control | | DLJ | | | | HLJ | | | FHLJ | | | | |
|  | g | kcal | | g | kcal | g | | kcal | | g | | kcal | | |
| Ingredients |  |  |  |  |  |  |  |  |  |  |  |  |  |  |
| Casein | 200 | 800 | | 200 | 800 | 200 | | 800 | | 200 | | 800 | | |
| Corn starch | 150 | 600 | | 150 | 600 | 150 | | 600 | | 150 | | 600 | | |
| Sucrose | 500 | 2,000 | | 500 | 2,000 | 500 | | 2,000 | | 500 | | 2,000 | | |
| Cellulose | 50 | - | | 50 | - | 50 | | - | | 50 | | - | | |
| Corn oil | 50 | 450 | | 50 | 450 | 50 | | 450 | | 50 | | 450 | | |
| Mineral mixture | 35 | - | | 35 | - | 35 | | - | | 35 | | - | | |
| Vitamin mixture | 10 | 40 | | 10 | 40 | 10 | | 40 | | 10 | | 40 | | |
| DL-methionine | 3 | 12 | | 3 | 12 | 3 | | 12 | | 3 | | 12 | | |
| Choline bitartrate | 2 | - | | 2 | - | 2 | | - | | 2 | | - | | |
| Total | 1,000 | 3,902 | | 1,000 | 3,902 | 1,000 | | 3,902 | | 1,000 | | 3,902 | | |
| DLJ |  |  | | 10% dried  *L. japonica* | | |  | |  | |  | |  | |
| HLJ |  |  | |  |  | 10% heat-treated  *L. japonica* | | | | |  | |  | |
| FHLJ |  |  | |  |  |  | |  | | 0.6% FOS and  10% heat-treated  *L. japonica* | | | |  |

**Supplementary Table 1.** Diet compositions for the control and treatment groups.
Control: basal diet group, DLJ: basal diet + 10% dried *L. japonica,* HLJ: basal diet + 10% dried *L. japonica* heat-treated at 100℃ for 30 minutes, FHLJ: HLJ + 6% FOS.
